# Antioxidant, Anti-Inflammatory, and Analgesic Properties of Chemically Characterized Polyphenol-Rich Extract from *Withania adpressa* Coss. ex Batt

**DOI:** 10.3390/life13010109

**Published:** 2022-12-30

**Authors:** Ahmad Mohammad Salamatullah

**Affiliations:** Department of Food Science & Nutrition, College of Food and Agricultural Sciences, King Saud University, P.O. Box 2460, Riyadh 11451, Saudi Arabia; asalamh@ksu.edu.sa

**Keywords:** plants, natural products, free radicals, inflammation, medicinal, caffeic acid

## Abstract

The current work was undertaken to investigate the chemical composition, antioxidant, anti-inflammatory, and analgesic properties of a polyphenol-rich fraction from *Withania adpressa* Coss. ex Batt. After being extracted, the polyphenol-rich fraction was chemically characterized through use of high-performance liquid chromatography (HPLC). Antioxidant potency was assessed through the use of 2,2-diphenyl-1-picrylhydrazyl (DPPH) and total antioxidant capacity (TAC). Inflammatory and analgesic properties were assessed in vivo through the use of carrageenan and heat stimulus assays, respectively. Chromatographic analysis of polyphenol-rich fraction revealed the presence of potentially bioactive phenols including epicatechin, apigenin, luteolin, quercetin, caffeic acid, p-coumaric acid, and rosmarinic acid. The polyphenol-rich fraction showed interesting anti-free-radical potency with a calculated IC_50_ value of 27.84 ± 1.48 µg/mL. At the highest dose used (1000 µg/mL), the polyphenol-rich fraction scored good total antioxidant capacity with a calculated value of 924.0 ± 28.29 µg EAA/mg. The polyphenol-rich fraction strongly alleviated the inflammatory effect of carrageenan injected into the plantar fascia of rats resulting in inhibition up to 89.0 ± 2.08% at the highest tested dose (500 mg/kg). The polyphenol-rich fraction showed a good analgesic effect wherein the delay in reaction time to a thermal stimulus caused by 500 mg/kg had a highly similar effect to that induced by Tramadol used as a positive control. The findings of the current work highlight the importance of polyphenol-rich fractions from *W. adpressa* Coss. ex Batt. as an alternative source of natural antioxidant, inflammatory, and analgesic drugs to control relative diseases.

## 1. Introduction

It is well known that plants have been utilized for medical reasons, cosmetic purposes, and as a dietary component all over the globe for hundreds of years [1,2]. The presence of phytochemicals in herbs, especially secondary metabolites, is the most important factor contributing to their beneficial characteristics [3]. Higher plants synthesize these organic molecules, which, in most instances, are not required for growth and development but are instead formed in reaction to biotic and abiotic environmental conditions [4]. In plants, secondary metabolites consist of terpenoids, alkaloids, and flavonoids, which have been scientifically shown to be promising bioactive agents with antioxidant, antibiotic, anti-inflammatory, anti-aging, and antitumor properties [5,6,7]. Depending on the use, plants may be used fresh, dried, or processed into essential oils or crude extracts [7]. The compounds responsible for particular biological potential in a plant have been examined in many studies [8].

Diets high in antioxidants have been shown to protect humans against degenerative illnesses including cancer and cardiovascular disease [9]. To avoid the oxidative degradation of foodstuffs caused by free radicals, natural antioxidants tend to be favored by users in the food business over synthetic antioxidants, according to recent research [10]. Free radicals have the potential to generate cytotoxic effects and tissue lesions, as well as DNA damage. The human body requires antioxidant agents in order to fight itself against free radicals. These antioxidant agents are found in fruits and vegetables and almost all plants. Because of their secondary metabolites, plants are able to supply potent antioxidant agents that help to manage and alleviate the effects of free radicals [11].

Inflammatory disorders are growing more widespread throughout the globe [12]. Inflammation may be triggered by several factors, including physical injury, ultraviolet irradiation, microbial invasion, and immunological responses. Sclerosis, inflammatory bowel disease, chronic asthma, and psoriasis are among disorders that may be caused by inflammation cascades. Inflammation is also involved in the development of chronic fatigue syndrome [13,14]. The disadvantages of clinically utilized anti-inflammatory medications include the presence of side effects as well as the high expense of treatment [12]. Traditional remedies and natural products may be used as an alternative to these treatments, and they hold significant promise in the discovery of bioactive lead compounds into therapeutics for the treatment of inflammatory illnesses. Traditional remedies and phytopharmaceuticals have been utilized for the treatment of inflammatory and other illnesses for many years [12].

*W. adpressa* Coss. ex Batt (*Solanaceae*), which is a herb commonly known by its name Winter Cherry, grows in North Africa and the Mediterranean basin; it has been shown to have pharmacological properties, including anti-tumor, immunomodulatory, anti-convulsant, and anti-stress properties. Importantly, diseases such as conjunctivitis, inflammation, anxiety, nervous system diseases, bronchitis, ulcers, liver disease, and Parkinson’s disease have been treated with plants in the genus of Withania for a long time [15]. Previous reports showed that the gnus *withania* possesses many phenols, notably glycowithanolides and withanolides, as well as volatile chemicals [16].

The current study was conducted to investigate the chemical composition, antioxidant, anti-inflammatory, and analgesic properties of a polyphenol-rich fraction from leaves *W. adpressa* Coss. ex Batt.

## 2. Materials and Methods

### 2.1. Plant Material

From the Sahara area (29.7519° N, 7.9756° W) in March 2021, *W. adpressa* Coss. ex Batt was collected. Following the confirmation of the plant’s identity by a botanist, it was placed in the University Herbarium under the reference A2/WDBF21. Consequently, the leaves were washed and dried for seven days in the dark and a well-ventilated environment before being extracted.

### 2.2. Extraction of Phenols

The extraction of phenols was successfully conducted by using maceration as previously described [17]. To summarize, a total of 100 g of *W. adpressa* leaves was macerated with 300 mL of methanol. Following that, the solvent was removed through the use of a rotary evaporator at decreased pressure and low temperature to obtain the concentrated extract. The obtained extract was solubilized in 500 mL of distilled water before extracting it three times further through the use of liquid–liquid extraction using 200 mL of each of the following solvents: hexane, chloroform, and ethyl acetate. Following that, the ethyl acetate layer was evaporated at decreased pressure using a rotary evaporator, which was used to remove the solvent. The residue was dissolved in 300 mL water once more and freeze-dried in order to obtain a polyphenol-rich fraction [12].

### 2.3. HPLC Analysis

The polyphenol-rich fraction was phytochemically characterized using the HPLC as described by Amrati [17], with minor modifications. The HPLC system was used for separation and identification of compounds. Polyphenol and standard samples were filtered using a 0.2 m membrane filter to eliminate particle residues before being injected. After that, a volume of 5 mL of polyphenol extract was injected over a C18 ZORBAX Eclipse column at an injection rate of 0.7 mL/min and a column temperature of 30 °C. Acidified water (acetic acid 0.1%) (A) and acetonitrile (B) were utilized as the mobile phase in this experiment, and the reaction was allowed to proceed for 65 min. Compounds were recognized by comparing their spectra to those of reference compounds under the same conditions.

### 2.4. In Vitro Antioxidant Activity of Polyphenol-Rich Fraction

In the current work, assessment of the antioxidant activity of polyphenol-rich extract was carried out through the use of DPPH and molybdate in triplicate assays.

#### 2.4.1. Antioxidant Power of Polyphenol-Rich Fraction Using DPPH Assay

This test was performed according to the methodology described by Kuramasamy et al. [18]. Briefly, 1000 µL of a methanolic DPPH (0.2 mM) solution was combined with the polyphenol-rich fraction (0–1 mg/mL). The obtained mixture was then held at room temperature for 30 min in the darkness, and the absorbance was measured at 517 nm. A blank solution consisting of 1000 µL of DPPH solution and 1000 µL of methanol was used to serve as a negative control. The blank solution, as well as samples and positive controls (quercetin and BHT), was produced under identical working conditions. Next, a spectrophotometer was used to quantify the absorbance decline, and the inhibition percentage was determined through the use of the following formula:

Inhibition (%) = [1 − (sample/control)] × 100

#### 2.4.2. Total Antioxidant Capacity of Polyphenol-Rich Fraction

A reagent was prepared by mixing H2SO4, (0.6 M), Na2PO4 (28 mM), and ammonium molybdate (4 mM) to measure the total antioxidant capacity of the polyphenol-rich fraction. Briefly, one milliliter of reagent was added to 0.1 mL of the polyphenol-rich fraction at various concentrations (0.2, 0.5, and 1 mg/mL). Thereafter, a blank solution composed of 1 mL reagent and 0.1 mL methanol was incubated in a water bath set to 95 °C for 90 min. Next, the absorbance was measured at 695 nm. The results were expressed in mg ascorbic acid equivalent per gram of dry extract (µg EAA/mg) [19].

### 2.5. Animal Material

Male rats weighing between 100 and 150 g were during the two-week acclimatization period; the animals were housed in cages with five rats each and maintained at 22 °C with a 12 h light–dark cycle. The method used in the present study complied with the globally recognized Guide for the Care and Use of Laboratory Animals. The animals were given unrestricted access to food and water at all times [20].

### 2.6. Anti-Inflammatory Activity

The anti-inflammatory activity of the polyphenol-rich fraction was assessed as described in earlier work. Animals were divided into groups of five rats each, of which two groups served as negative and positive controls, which received 0.9% saline and Diclofenac (1%), respectively, while other groups served as treatments, which received the polyphenol-rich fraction. Ninety minutes after local applications or one hour after oral administration of the polyphenol-rich fraction at different doses (200, 400, and 500 mg/kg), the plantar fascia of the right hind leg of rat was injected with 0.1 mL of carrageenan intradermally. The circumference of the applied sample was measured before the injection of carrageenan and then after every hour from the third hour until the sixth hour after the administration of carrageenan. The following formula was used to compute the % inhibition of inflammation:

% inhibition= [((S_t_ − S_0_) control − (S_t_ − S_0_) sample)/((S_t_ − S_0_) control)] × 100

### 2.7. Analgesic Activity

The animals were divided into groups of five rats each, of which two groups served as negative and positive controls, which received 0.9% saline and Tramadol, respectively. Each animal was individually placed in an enclosed space on a glass surface (L × W × H = 10 × 20 × 14). After 10 min of adaptation, rats received oral administration of polyphenol-rich fraction before being subjected to the heat stimulus (50 °C) onto the plantar surface of each hindpaw. The increase in temperature under the plantar fascia of the right hind leg resulted in rat movement. The delay in reaction time to the thermal stimulus was recorded.

### 2.8. Statistical Analysis

Data were expressed in means with standard deviations of triplicate tests using GraphPad Prism software (version.7). Normality of distributions was tested by the use of Shapiro–Wilk’s test, whilst the homogeneity of variances was checked by the use of Levene’s test. Analysis of variance (ANOVA) was performed, with Tukey’s HSD test as a post hoc test for multiple comparisons. A significant difference was considered at *p* < 0.05.

## 3. Results

### 3.1. Chemical Characterization

**Figure 1:**
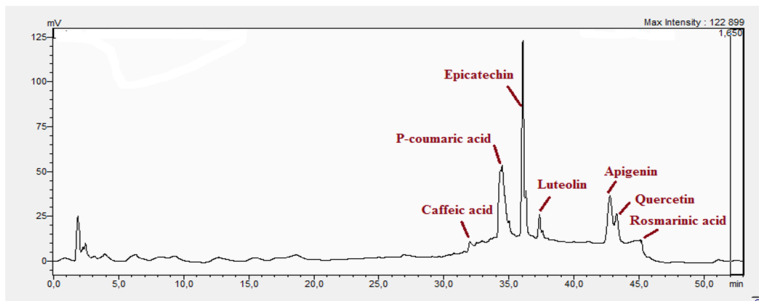
Chromatographic analysis of polyphenol-rich fraction using HPLC.

The chemical characterization of the polyphenol-rich fraction from leaves of *W. adpressa* allowed the identification of seven major compounds including flavonoid compounds; epicatechin, apigenin, luteolin, and quercetin; phenolic acids; caffeic acid and p-coumaric acid; and polyphenols derived from hydroxycinnamic-acid-like rosmarinic acid (Figure 1, Table 1). The chemical composition of different extracts from the genus Withania has been widely investigated. Notably, Jain et al. (2012) revealed that extracts from *Withania somnifera* and *Withania coagulans* possessed alkaloids; isopelletierine, anaferine, and saponins with an additional acyl group; sitoindoside VII and VIII; withanolides with glucose at carbon 27; withanolides; and withaferines [21]. Matsuda and co-authors (2001) showed that *W. somnifera* possessed withanolide glycosides and withanosides [22]. The genus Withania possessed various fatty acids; octacosan; oleic and stearic fatty acids; steroids; and oleanolic acid as reported in earlier work [23].

### 3.2. Antioxidant Activity

**Figure 2:**
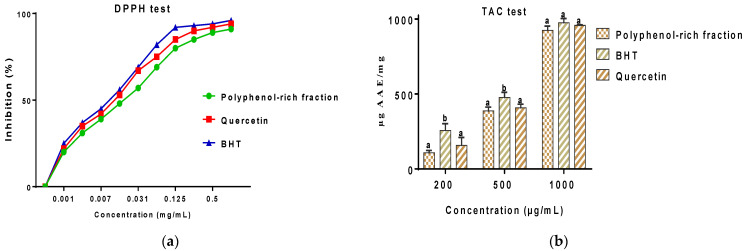
Antioxidant activity of polyphenol-rich fraction by the use of DPPH (**a**) and ammonium molybdate (**b**). The graphs have the same letter do not present a significant difference.

The antioxidant activity of the polyphenol-rich fraction using the DPPH method, as represented in Figure 1, showed that the polyphenol-rich fraction exhibited good antioxidant activity in a dose-dependent manner. From this figure, it can be seen that increasing the concentration of the polyphenol-rich fraction increased the inhibition percentage of DPPH free radicals, i.e., 10 and 100 µg/mL of the polyphenol-rich fraction inhibited 42% and 72%, respectively (Table 1). The polyphenol-rich fraction recorded an IC_50_ value of 27.84 ± 1.48 µg/mL, which can be considered important when compared to that obtained with BHT (13.42 ± 0.87 µg/mL) and quercetin (14.27 ± 0.59 µg/mL) (Table 2). Antioxidant capacity evaluated by the use of ammonium molybdate showed that the polyphenol-rich fraction possessed important antioxidant capacity as shown in (Figure 2b). From this figure, it can see that 1000 µg/mL of the polyphenol-rich fraction recorded antioxidant capacity in the order of 924.0 ± 28.29 µg EAA/mg and 500 µg/ml recorded antioxidant capacity in the order of 387.1 ± 25.45 µg EAA/mg (Figure 2b).

The results of antioxidant activity showed that the polyphenol-rich fraction possessed excellent antioxidant power, which can be explained by its richness in phenols with antioxidant power such as epicatechin, apigenin, luteolin, quercetin, caffeic acid, p-coumaric acid, and rosmarinic acid (Figure 1). Previous work on caffeic acid (phenolic acid) revealed an inhibition percentage of DPPH radicals in the order of 93.9% (DPPH), even at a low concentration (20 µg/mL) [24]. This antioxidant power may be due to the richness of polyphenol-rich fractions in polyphenols, which could react with free radicals, whether separately or in synergy, resulting in an antioxidant effect [25]. Catechin and epicatechin contained in the polyphenol-rich fraction are the predominant compounds with antioxidant power as reported in earlier work [26]. Our findings are in agreement with those reported by El Moussawi and co-authors who showed that the genus Withania possessed antioxidant potency, particularly *Withania frutescens*, which revealed an anti-free-radical activity of the order of 477.65 µg EAA/mg [27].

### 3.3. Anti-Inflammatory Activity of Polyphenol-Rich Fraction

**Figure 3:**
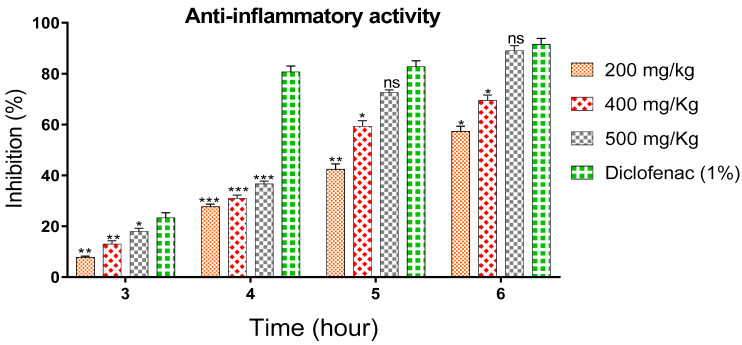
The anti-inflammatory activity of polyphenol-rich fraction at different doses (200, 400, and 500 mg/Kg) and Diclofenac (1%). *p* ≤ 0.05 (*); *p* ≤ 0.005 (**); *p* ≤ 0.001 (***); ns: no significant difference.

The anti-inflammatory activity of the polyphenol-rich fraction was studied by measuring the amount of edema induced by carrageenan in rats. The results are presented in Figure 3, which shows the percentage of anti-inflammatory evolution of edema as a function of time. From this table, it can be seen that the polyphenol-rich fraction given to mice via oral administration alleviated the inflammatory effect of carrageenan injected into the plantar fascia of the right posterior leg of rats within three hours following injection, resulting in inhibition of 13.02 ± 1.27 % at 400 mg/kg and 18 ± 1.20% at 500 mg/kg. The anti-inflammatory effect increased progressively and reached a maximum inhibition after six hours of post-treatment, which reached 57.32 ± 2.05% at 200 mg/kg, 69.46 ± 2.13% at 400 mg/kg, and 89. 0 ± 2.08% at 500 mg/kg, while the positive control (Diclofenac 1%) inhibited edema by 91.51 ± 2.41% (Figure 3). The pretreatment of rats with the different doses of polyphenol-rich fraction induced a strong inhibition of inflammation at the sixth hour when compared to Diclofenac used as a drug reference.

Carrageenan injection triggers an increase in the levels of cyclooxygenase 2 (COX-2) mRNA synthesis, resulting in an increased concentration of this enzyme, which peaks at 1 h [28]. This is accompanied by an increase in the synthesis of prostaglandins (PGs), mainly prostaglandin E2 (PGE2) (maximum at 2 h) which is mainly involved in certain pain and inflammation processes [29]. This feature helps explain why non-steroidal anti-inflammatory drugs (NSAIDs), such as aspirin, have no effect at 1 h. This delay is due to their mechanisms of operation, namely the inhibition of PGs by simultaneously stimulating the two enzymes COX-1 and COX-2, for which the inhibition curve consolidates after 3 h, reflecting the stabilization of the mediators [30]. As for steroidal anti-inflammatory drugs (AIS) such as dexamethasone, their action is apparent from the first hour, thanks to their direct interaction with DNA, whose effect is associated with the action of several molecular pathways including pro-inflammatory cytokines, phospholipase, and COX, mainly through the nuclear transcription factor NF-kB [30,31].

In the present work, the anti-inflammatory effect of the polyphenol-rich fraction by the carrageenan-induced edema test showed interesting results, whereby the inflammation was reduced by 89% at 500 mg/kg. These results are in agreement with those reported by Elmoussaoui et al. [29], who revealed 82.20% ± 8.69 as a percentage of inhibition induced by 450 mg/kg *Withania frutescens* extract [29]. These results could be explained by various polar phenolic compounds identified by HPLC in polyphenol-rich fractions such as epicatechin, apigenin, luteolin, quercetin, caffeic acid, p-coumaric acid, and rosmarinic acid. Other phytoconstituents in the genus Withania, particularly tannins, mucilages, alkaloids, coumarins, and free quinone, could be also responsible for the anti-inflammatory activity investigated in the present work. These compounds can act by preventing the synthesis of prostaglandins through cyclooxygenase [14,17,32,33].

*Withania somnifera* exerted an anti-inflammatory role by repressing the expression of certain cytokines including tumor necrosis factor-(TNF-) α, interleukin-(IL-) 8 and 1, nitric oxide, and reactive oxygen species. Leukocyte adhesion and migration, as well as cell adhesion molecules, the production of IL-6 and TNF-a, and the activation of NF-k (nuclear factor kappa-luminous chain-enhancer of activated B-cells), were successfully blocked by withaferin A, one of the active components in *W. somnifera*. [18,20]. In addition, this compound blocked the activation of PMA-induced phosphorylation of the transcription factor p38, extracellular-regulated kinases (ERK 12), and the transcription factor c-Jun N terminal kinase (c-Jun N-terminal kinase) (JNK) [18,21].

### 3.4. Analgesic Activity of Polyphenol-Rich Fraction

**Figure 4:**
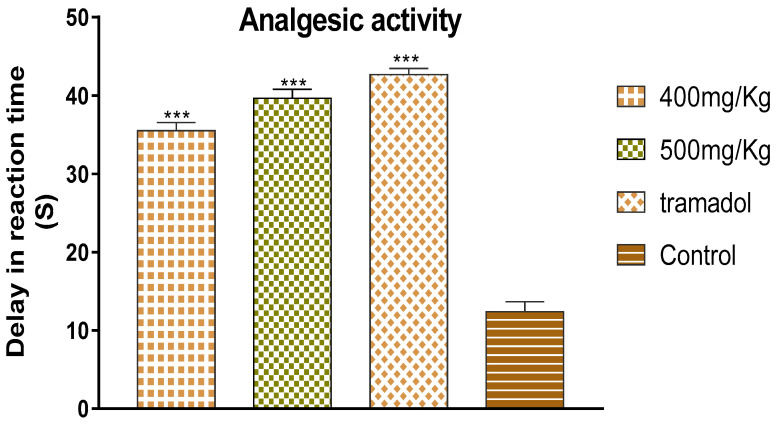
Analgesic activity of polyphenol-rich fraction. *p* *** ≤ 0.001.

Regarding analgesic activity, Figure 4 provides the result of the paw withdrawal test performed using the plantar thermal hyperalgesia model. The results showed that the paw withdrawal latency of the animal treated with the polyphenol-rich fraction was significantly higher than that of the negative control group which received only 0.9% NaCl physiological solution. The delay in reaction time to the thermal stimulus was directly related to the increase in doses of the sample. Notably, the delay in reaction time for 400 mg/kg and 500 mg/kg was 35.51 ± 1.04 s and 39.64 ± 1.17 s, respectively, while the delay in reaction time for the negative control group was 12.38 ± 1.28 s. Rats treated with Tramadol (1%), the reference analgesic, had a longer delay in reaction time to the thermal stimulus than the groups treated with the different doses of the polyphenol-rich fraction, which was 42.68 ± 0.78 s.

The results show that the delay in reaction time to the thermal stimulus caused by 500 mg/kg of the polyphenol-rich fraction had a similar effect to that induced by the drug used as a positive control. This result can be considered a strong indication of the analgesic effect of the plant extract. The analgesic effect of the polyphenol-rich fraction can be explained by the property of phytochemicals identified using HPLC without excluding other phytochemicals in the genus Withania such as withanolides, withaferin A, withanolide F, coagulin L, and nicotiflorin, which are known to possess a notable anti-nociceptive property [34,35,36,37,38]. All these results are in agreement with those reported by Moussaoui et al. (2020) who revealed an important analgesic effect of the methanolic extract of *Withania frutescens* leaves at the dose of 450 mg/kg using the acetic acid method [29]. In addition, the current study was in accordance with Kumar et al. (2015), who demonstrated that *Withania somnifera* extract significantly increased the potency and time of the pain threshold as well as the potency and time of pain tolerance compared to placebo, reflecting a significant analgesic effect [39,40]. The analgesic effect presented here can be explained by the fact that phenols exhibited a central anti-nociceptive effect via the activation of opioid receptors whose activation results in a decrease in the release of pain mediators such as substance P [5,22].

## 4. Conclusions

The current work highlighted the antioxidant, anti-inflammatory, and analgesic properties of chemically characterized polyphenols from *Withania adpressa* Coss. This study concluded that polyphenols from *Withania adpressa* Coss. possess a potential for being used as an alternative reservoir of natural antioxidant, inflammatory, and analgesic drugs to control relative diseases. Even though the outcome of the present work is highly interesting, further pre-clinical and clinical investigations on nonhuman primates and humans will be required before any possible use of polyphenol-rich fractions as a natural drug.

## Figures and Tables

**Table 1:** Chemical structure of major compounds identified in polyphenol-rich fraction using HPLC.

RT	Identification Compound	Standard Use	Concentration in µg/mg	Molecular Structure
31.91	Caffeic acid	Cafeic acid	71.28	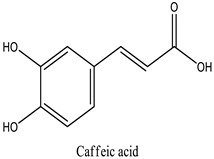
34.76	Coumaric acid	p-coumaric acid	30.46	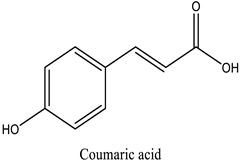
36.09	Epicatechin	Epicatechin	40.53	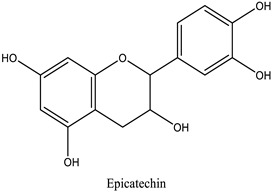
37.41	Chrysoeriol-7-diglucuronide	Luteolin	33.62	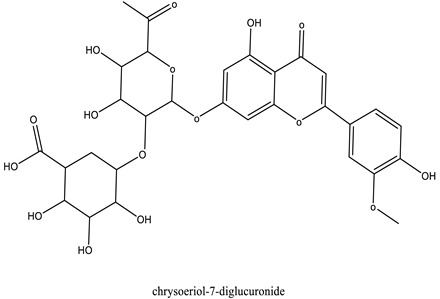
42.78	Acacetin-7-diglucuronide	Apigenin	34.20	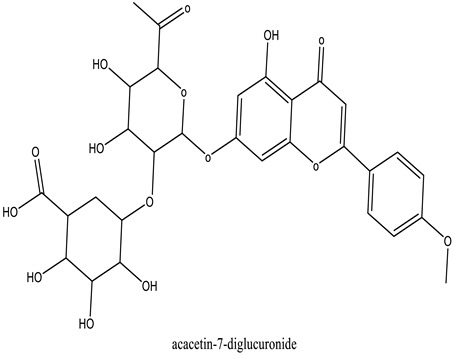
43.35	Quercetin-3-*O*-glucuronide	Quercetin	64.25	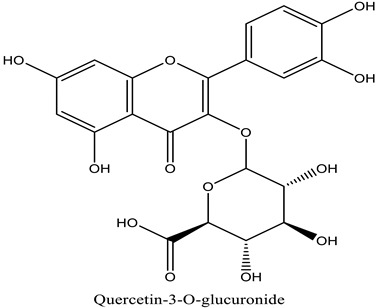
45.19	Rosmarinic acid	Rosmarinic acid	39.81	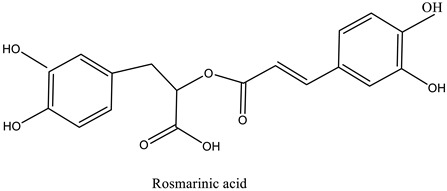

**Table 2:** Antioxidant power of polyphenol-rich fraction tested by the use of DPPH bioassay.

Samples	Anti-Radical Activity by the DPPH Method			IC-50 in µg/mL
	10 µg/mL	100 µg/mL	1000 µg/mL	
Polyphenol-rich fraction	42%	72%	91%	27.84 ± 1.48
Quercetin	46%	87%	94%	14.27 ± 0.59
BHT	48%	89%	96%	13.42 ± 0.87

## Data Availability

Not applicable.
